# Leptospirosis, melioidosis, and rickettsioses in the vicious circle of neglect

**DOI:** 10.1371/journal.pntd.0012796

**Published:** 2025-01-23

**Authors:** Tshokey Tshokey, Ablert I. Ko, Bart J. Currie, Claudia Munoz-Zanzi, Cyrille Goarant, Daniel H. Paris, David A. B. Dance, Direk Limmathurotsakul, Emma Birnie, Eric Bertherat, Gyanendra Gongal, Jackie Benschop, Jelmer Savelkoel, John Stenos, Kartika Saraswati, Matthew T. Robinson, Nicholas P. J. Day, Stephen R. Graves, Steven R. Belmain, Stuart D. Blacksell, Willem J. Wiersinga

**Affiliations:** 1 Australian Rickettsial Reference Laboratory, University Hospital Geelong, Geelong, Australia; 2 Department of Pathology and Laboratory Medicine, Jigme Dorji Wangchuck National Referral Hospital, Thimphu, Bhutan; 3 Department of Epidemiology of Microbial Diseases, Yale School of Public Health, New Haven, Connecticut, United States of America; 4 Instituto Gonçalo Moniz, Fundaçao Oswaldo Cruz, Salvador, Brazil; 5 Menzies School of Health Research, Charles Darwin University and Royal Darwin Hospital, Darwin, Australia; 6 Division of Environmental Health Sciences, School of Public Health, University of Minnesota, Minneapolis, Minnesota, United States of America; 7 The Pacific Community, Public Health Division, Noumea, New Caledonia; 8 Department of Medicine, Swiss Tropical and Public Health Institute, Allschwil, Switzerland; 9 Department of Clinical Research, University of Basel, Basel, Switzerland; 10 Lao-Oxford-Mahosot Hospital-Wellcome Trust Research Unit. Microbiology Laboratory, Mahosot Hospital, Vientiane, Lao People’s Democratic Republic; 11 Faculty of Infectious and Tropical Diseases, London School of Hygiene and Tropical Medicine, London, United Kingdom; 12 Mahidol Oxford Tropical Medicine Research Unit, Faculty of Tropical Medicine, Mahidol University, Bangkok, Thailand and Centre for Tropical Medicine and Global Health, Nuffield Department of Medicine, University of Oxford, Oxford, United Kingdom; 13 Department of Tropical Hygiene, Faculty of Tropical Medicine, Mahidol University, Bangkok, Thailand; 14 Centre for Tropical Medicine and Global Health, Nuffield Department of Medicine, University of Oxford, Oxford, United Kingdom; 15 Center for Infection and Molecular Medicine, Amsterdam UMC location University of Amsterdam, Amsterdam, the Netherlands; 16 Division of Infectious Diseases, Department of Medicine, Amsterdam UMC location University of Amsterdam, Amsterdam, the Netherlands; 17 Groupe d’Intervention en Santé Publique, Marseille, France; 18 World Health Emergency Programme, WHO Regional Office for South-East Asia, New Delhi, India; 19 Tāwharau Ora, School of Veterinary Science, Massey University, Palmerston North, New Zealand; 20 Saw Swee Hock School of Public Health, National University of Singapore, Singapore, Republic of Singapore; 21 Oxford University Clinical Research Unit Indonesia, Faculty of Medicine, Universitas Indonesia, Jakarta, Indonesia; 22 Natural Resources Institute, University of Greenwich, London, United Kingdom; University of Kentucky College of Medicine, UNITED STATES OF AMERICA

## Abstract

The global priorities in the field of infectious diseases are constantly changing. While emerging viral infections have regularly dominated public health attention, which has only intensified after the COVID-19 pandemic, numerous bacterial diseases have previously caused, and continue to cause, significant morbidity and mortality—deserving equal attention. Three potentially life-threatening endemic bacterial diseases (leptospirosis, melioidosis, and rickettsioses) are a huge public health concern especially in low- and middle-income countries. Despite their continued threat, these diseases do not receive proportionate attention from global health organizations and are not even included on the WHO list of neglected tropical diseases (NTDs). This, in turn, has led to a vicious circle of neglect with continued, yet conceivably preventable, hospitalizations and deaths each year especially in the vulnerable population. This is a call from a group of multi-institutional experts on the urgent need to directly address the circle of neglect and raise support in terms of funding, research, surveillance, diagnostics, and therapeutics to alleviate the burden of these 3 diseases.

## Background

Epidemic and pandemic-prone viral diseases, the most recent example being COVID-19 (SARS-CoV-2), have been at the forefront of global concerns, leading to systematic efforts to improve surveillance and identify interventions. However, to date, largely endemic bacterial diseases, some of which have epidemic potential, still represent a significant source of Public Health Emergencies of International Concern (PHEIC). Beyond that, bacterial diseases in their endemic presentation are a considerable burden on the health and well-being of the global population.

Leptospirosis, melioidosis, and rickettsioses represent perfect examples of this situation. Most often affecting the most vulnerable populations, these diseases can be qualified as “forgotten diseases of neglected populations.” These 3 diseases have a substantial estimated global burden disproportionately affecting the poor, marginalized, and hard-to-reach population in low- and middle-income countries (LMICs) who are already burdened with multiple medical, social and economic tribulations. Similarities among these diseases include environmental reservoirs, social determinants affecting disease outcomes, and specific at-risk populations. It is not uncommon that the 3 diseases are simultaneously hyperendemic in the same country such as the tropical countries in Southeast Asia. Environmental conditions and alterations resulting from climate change, urbanization, and land use changes substantially influence transmission risk and increased population vulnerability for all 3 diseases.

Further, they fall under the umbrella of acute undifferentiated febrile illnesses that require laboratory support for proper diagnosis. Often, facilities are missing or are insufficiently equipped to establish a microbiologically confirmed diagnosis, which also hinders epidemiological surveillance efforts to inform the local disease burden. Leptospirosis, melioidosis, and rickettsioses are key examples of diseases affected by a lack of awareness and systematically underdeveloped surveillance efforts, which are needed for innovative research and control strategies **(Table 1).**

**Table 1:** Brief overview of leptospirosis, melioidosis, and rickettsioses.

	Leptospirosis	Melioidosis	Rickettsioses
**Etiology**	Pathogenic *Leptospira* species. Species that most often are associated with severe infections include *L*. *interrogans*, *L*. *kirschneri*, *L*. *noguchii*.	Gram-negative bacterium *Burkholderia pseudomallei*	Gram-negative bacteria in the family *Rickettsiaceae*, of which scrub typhus, murine typhus, spotted fever group (SFG) typhus, and epidemic typhus are of special interest.
**Epidemiology**	Global distribution with higher levels of endemicity in (sub)tropical regions	Global and expanding (sub)tropical distribution	- Scrub typhus: Asia and Pacific (traditionally in the *“Tsutsugamushi triangle”)*, with increasing evidence of wider distribution [5] - Murine typhus, SFG typhus: global distribution - Epidemic typhus: South America, Africa, USA (rare)
**Estimated incidence and mortality**	1,030,000 cases and 58,900 deaths/year [6]	165,000 cases and 89,000 deaths/year [2,7]	- Scrub typhus [8]: approx. 1,000,000 cases/year; median mortality 6.7% in hospitalized patients - Murine typhus, SFG typhus: Unknown - Epidemic typhus: Currently low but epidemic potential
**Estimated disease burden**	2.9 million DALYs/year [9]	4.6 million DALYs/year [7]	Not yet estimated in terms of DALYs
**Transmission**	Zoonotic and environmental acquisition via inoculation or mucous membranes	Environmental acquisition via inoculation, inhalation, or ingestion	Arthropod-borne
**Risk factors**	Occupational exposure (e.g., farmers, fisherman, sewage workers), domestic (e.g., contact with water and soil), and recreational exposure (e.g., watersports), severe weather events (e.g., heavy rainfall, flooding)	Diabetes, chronic lung, liver and kidney diseases, hazardous alcohol use, immunosuppressive therapy occupational exposure (e.g., farmers), severe weather events (e.g., heavy rainfall, flooding)	Scrub typhus: occupational exposure (e.g., farmers, military personnel) Murine typhus: rat infestations SFG typhus: outdoor activities (e.g., camping, safaris), tick abundance, and exposure in endemic areas Epidemic typhus: conflicts, refugee camps
**Clinical presentation**	Nonspecific febrile illness to Weil’s disease, renal and cardiac failures, or severe pulmonary hemorrhage	Localized skin lesions to abscesses (e.g., liver, spleen, prostate), pneumonia, and bacteremia with severe sepsis	Nonspecific febrile illness to fatal complications, with acute respiratory distress syndrome, multiple organ dysfunction syndrome, and meningo-encephalitis
**Diagnosis–gold standard**	Culture, (q)PCR, microscopic agglutination test	Culture	Serology by IFA
**Treatment**	Doxycycline, amoxicillin, ampicillin or i.v. penicillin G, third generation cephalosporins or erythromycin	i.v. meropenem or i.v. ceftazidime followed by oral trimethoprim/sulfamethoxazole	Oral doxycycline (azithromycin in pregnancy and children); i.v. doxycycline and i.v. azithromycin in severe cases
**Prevention**	Protective equipment (e.g., shoes, gloves), basic hygienic measures, clean food and drinking water, vaccination of domestic animals, rodent control	Advice on avoidance of exposure especially for those with clinical risk factors (e.g., diabetes). Protective equipment (e.g., shoes, gloves), basic hygienic measures, and treated drinking water	Protective equipment (e.g., long sleeves), vector control, insecticides, basic hygienic measures

DALY, disability adjusted life year; i.v., intravenous; ELISA, enzyme-linked immunosorbent assay; IFA, indirect immunofluorescence assay; NA, not available.

## A vicious circle of neglect

These 3 diseases are neglected by international institutions and policy-makers, leading to a vicious cycle of continued neglect. Adequate attention and funding from international institutions and donors do not match the observed needs, despite their significant burdens, current and future predictions regarding climate and socioeconomic changes, and concerns expressed in most endemic countries [1,2]. The estimated global disease burdens of leptospirosis and melioidosis compared to the diseases officially classified as neglected tropical diseases (NTDs) by the WHO justifies their inclusion [3] **(Table 2)**.

**Table 2:** Comparative burdens of leptospirosis and melioidosis, and the diseases officially classified as NTD.

Disease	Number of cases (millions)	Deaths	DALYs (millions)
**Leptospirosis** [6]	**1.03**	**58,900**	**2.90**
**Melioidosis** [7]	**0.165**	**89,000**	**4.64**
Intestinal nematode infections [3]	909	2,090	1.97
Visceral leishmaniasis [3]	0.03	5,710	0.40
Schistosomiasis [3]	140	11,500	1.64
Lymphatic filariasis [3]	71.9	-	1.63
Food-borne trematodiasis [3]	33.5	-	0.78
Rabies [3]	0.01	13,700	0.78
Dengue [3]	56.9	36,100	2.38

In the same way, the World Health Organization’s Research and Development (WHO R&D) Blueprint, whose objective is to promote the development of countermeasure tools against priority epidemic diseases, has so far limited its road maps to viral diseases [4]. This neglects the risk associated with bacterial endemic and epidemic diseases, which is increasing with the alarming development of antimicrobial resistance and vaccine hesitancy. Leptospirosis, with its epidemic potential associated with natural catastrophes and the absence of efficient diagnostic and countermeasure tools, is a perfect example of a disease that should benefit from the R&D Blueprint. Moreover, the international partners that align with the WHO tend to (wrongly) assume that diseases not on the NTD list are not neglected and are already receiving the attention and funding they need. The situations of melioidosis and rickettsiosis are no better than leptospirosis.

The lack of investment in R&D is at the origin of a cascade of consequences increasing the disease burden. The absence of practical and accurate laboratory diagnostic tools results in less attention being given to these diseases in public health and clinical practice leading to misdiagnoses and inappropriate treatment of patients. This also leads to the absence of these diseases from routine epidemiological surveillance and a lack of data. Consequently, there is a failure to incentivize R&D investment and to develop target product profiles. Another downside is that when research is under-resourced, the field becomes unattractive for junior researchers, and eventually, the international expert community shrinks.

Meanwhile, neglect of these diseases results in a lack of guidelines, infrequent training events for health professionals, and the absence of a global prevention and control strategy. Efforts are made to educate the at-risk populations, but in practice, health authorities have few practical and effective solutions to protect people. In this context, the importance of vector and rodent control for certain pathogens must also be underlined. While diseases transmitted by mosquitos have benefited from intense mosquito control efforts for decades, this is not the case for most of the other potential vector-borne diseases, such as those transmitted by ticks, fleas, lice, or mites. The development of insecticide resistance is alarming, and the number of people specialized in vector control is rapidly diminishing. The same applies to diseases transmitted by rodents.

Leptospirosis, melioidosis, and rickettsioses can mimic infections caused by other highly pathogenic agents, but they are not systematically investigated in the field during potential public health events of international importance; laboratory investigations too often focus solely on viral pathogens. The absence or delay of an appropriate diagnosis increases morbidity and mortality, which might otherwise have been avoided. When finally recognized, the field response to these diseases is suboptimal because of a lack of preparedness and clear control strategies. These inadequate responses lead to preventable morbidity and mortality. An ongoing event itself might raise attention within the international community and mobilize some funding. However, this is limited to that one event, while long-term investment is needed to improve the prevention and control of these diseases. The vicious circle of neglect is perpetuated due to poor data when the events occur in LMICs that are unable to conduct proper surveillance, diagnostic investigations, and research.

## Advocacy and opportunities for innovation and breakthroughs

How might we fill in the gap between the concerns of the endemic countries and the agenda of the international institutions? Now is a crucial time to focus attention on these 3 neglected diseases. This is largely attributed to, and might partly be addressed by, the advances in technology and methodologies that are now at our disposal which could significantly improve various aspects of these diseases, from laboratory diagnosis to eco-epidemiological research. Giving greater priority to these diseases now would mean a good return on investment. We can provide examples of how to enhance disease surveillance and prevention. For example, we can utilize genotyping and genomic analysis for monitoring purposes. Additionally, the various diagnostic “fever panel” platforms developed in response to COVID-19 could be strategically expanded to include these and other bacterial diseases. Furthermore, the latest generation of vaccines could be adapted for these diseases, allowing for a more tailored prevention strategy. It will also mandate diagnostics and vaccines to be more widely available and affordable since the endemicity of these diseases with epidemic potential mostly affect the developing nations with limited resources.

Although an established concept, One Health [10] awareness has increased considerably in recent years due to the need for a comprehensive approach to dealing with diseases of epidemic or pandemic potential. Current research and public health environments are more prepared to work in this perspective; more people are sensitized to One Health, and more programs are being established on this platform with cross-disciplinary language and methods. Leptospirosis, melioidosis, and rickettsioses are prime examples of One Health diseases because of their complex transmission cycles, including both zoonotic and environmentally driven routes, and the requirement for a suitable multidisciplinary and multi-sectoral approach to plan for and respond to outbreaks and epidemics. However, the political mobilization relating to One Health and the associated funding streams have not benefited these emblematic diseases so far. Let us hope that they will in the future.

## References

[1] Salje J, Weitzel T, Newton PN, Varghese GM, Day N. Rickettsial infections: A blind spot in our view of neglected tropical diseases. PLoS Negl Trop Dis. 2021;15(5). doi: 10.1371/journal.pntd.0009353 33983936 PMC8118261

[2] Savelkoel J, Dance DAB, Currie BJ, Limmathurotsakul D, Wiersinga WJ. A call to action: time to recognise melioidosis as a neglected tropical disease. Lancet Infect Dis. 2022;22. 34953519 10.1016/S1473-3099(21)00394-7

[3] Global Health Data Exchange. Global Burden of Disease Study 2019 (GBD 2019) Data Resources | GHDx [Internet]. Available from: https://ghdx.healthdata.org/gbd-2019.

[4] World Health Organization (WHO), Geneva, Switzerland. WHO R&D blueprint [Internet]. [cited 2024 Oct 15]. Available from: https://www.who.int/observatories/global-observatory-on-health-research-and-development/analyses-and-syntheses/who-r-d-blueprint/background.

[5] Luce-Fedrow A, Lehman ML, Kelly DJ, Mullins K, Maina AN, Stewart RL, et al. A Review of Scrub Typhus (Orientia tsutsugamushi and Related Organisms): Then, Now, and Tomorrow. Trop Med Infect Dis [Internet]. 2018 Jan 17 [cited 2024 Oct 21];3(1). Available from: https://pubmed.ncbi.nlm.nih.gov/30274407/. doi: 10.3390/tropicalmed3010008 30274407 PMC6136631

[6] Costa F, Hagan JE, Calcagno J, Kane M, Torgerson P, Martinez-Silveira MS, et al. Global Morbidity and Mortality of Leptospirosis: A Systematic Review. PLoS Negl Trop Dis. 2015;9(9). doi: 10.1371/journal.pntd.0003898 26379143 PMC4574773

[7] Birnie E, Virk HS, Savelkoel J, Spijker R, Bertherat E, Dance DAB, et al. Global burden of melioidosis in 2015: a systematic review and data synthesis. Lancet Infect Dis. 2019;19(8). doi: 10.1016/S1473-3099(19)30157-4 31285144 PMC6867904

[8] Bonell A, Lubell Y, Newton PN, Crump JA, Paris DH. Estimating the burden of scrub typhus: A systematic review. PLoS Negl Trop Dis. 2017;11(9). doi: 10.1371/journal.pntd.0005838 28945755 PMC5634655

[9] Torgerson PR, Hagan JE, Costa F, Calcagno J, Kane M, Martinez-Silveira MS, et al. Global Burden of Leptospirosis: Estimated in Terms of Disability Adjusted Life Years. PLoS Negl Trop Dis. 2015;9(10). doi: 10.1371/journal.pntd.0004122 26431366 PMC4591975

[10] Adisasmito WB, Almuhairi S, Behravesh CB, Bilivogui P, Bukachi SA, Casas N, et al. One Health: A new definition for a sustainable and healthy future. PLoS Pathog. 2022;18(6):e1010537. doi: 10.1371/journal.ppat.1010537 35737670 PMC9223325

